# Regulation of *WOX11* Expression Represents the Difference Between Direct and Indirect Shoot Regeneration

**DOI:** 10.3389/fpls.2022.850726

**Published:** 2022-03-04

**Authors:** Jiong Hui Liu, Wan Chen Dong, Fang Fang Fei, Xiao Tong Li, Xiao Hang Zhang, Yangyan Zhou, Xian Sheng Zhang, Ya Lin Sang, Zhi Juan Cheng

**Affiliations:** ^1^State Key Laboratory of Crop Biology, State Forestry and Grassland Administration Key Laboratory of Silviculture in Downstream Areas of the Yellow River, College of Life Sciences, College of Forestry, Shandong Agricultural University, Taian, China; ^2^Shandong Salver Group, Salver Academy of Botany, Rizhao, China

**Keywords:** shoot regeneration, meristem, callus, lateral root primordial, *WOX11*, DNA methylation

## Abstract

Somatic cells of higher plants possess the remarkable ability to regenerate new individuals via reestablishing apical meristems. Reconstitution of shoot meristem is the vital process and is required for application of plant biotechnology. Under *in vitro* culture condition, shoot meristem can be formed directly or indirectly, depending on the absence or presence of callus as the intermediate status. However, the difference of regulatory mechanisms between the two regeneration types remains unknown. In this study, we established a bi-directional system in which shoots regenerated directly from lateral root primordia (LRP) and indirectly from hypocotyl-derived callus simultaneously. The results based on this system revealed that regulation of *WOX11* expression represents the difference between the two regeneration types in two aspects. Firstly, number of founder cells expressing *WOX11* is tightly associated with regeneration types. Relatively more founder cells gave rise to callus and produce larger meristem, whereas less founder cells produce LRP that regenerate smaller meristem. Secondly, non-CG DNA methylation specifically regulated *WOX11* transcription in LRP and promoted direct shoot regeneration, but had no influence on indirect regeneration. The results provide new insights for understanding the regulatory mechanisms of cell fate transition during *de novo* organogenesis.

## Introduction

Plant somatic cells have a powerful capacity to generate whole individuals under *in vitro* conditions (Su et al., 2011). A normal process is *de novo* organogenesis, in which the explants give rise to ectopic meristems and subsequently shoots and roots. The balance of phytohormones auxin and cytokinin controls the developmental types of regenerating organs. High ratios of auxin to cytokinin induced root formation, whereas low ratios of auxin and cytokinin led to shoot regeneration (Skoog and Miller, 1957). *De novo* organogenesis is the prerequisite of micropropagation and genetic transformation, and provide an important system for studying fundamental biological questions (Sang et al., 2018a; Williams and Garza, 2021).

Shoots can be induced from the explants directly or indirectly, which relies on absence or presence of callus, a mass of proliferating cells, in the intermediate phase (Ikeuchi et al., 2019). The callus for shoot regeneration originates from perivascular cells which are similar to the founder cells of lateral roots (Zhai and Xu, 2021). Different lines of evidences have shown that the founder cells do not undergo dedifferentiation but give rise to callus via a procedure similar to lateral root formation (Atta et al., 2009; Sugimoto et al., 2010). The callus could eventually generate roots or shoots depending on the concentration of auxin and cytokinin of the medium (Che et al., 2007). The typical example of direct regeneration is the conversion of lateral root primordia (LRP) to shoot meristems. Under induction of exogenous cytokinin, LRPs can be converted to shoot meristems without forming callus (Atta et al., 2009; Chatfield et al., 2013; Kareem et al., 2015; Rosspopoff et al., 2017). The conversion from LRPs to shoot meristems occurs within a narrow developmental window and is defined to be a transdifferentiation process.

*De novo* organogenesis comprises three steps. During the first step, auxin induces the transcription of *WUSCHEL-RELATED HOMEOBOX11/12* (*WOX11/12*), which encode homeodomain transcription factors, and promote the transition of perivascular cells to founder cells (Liu et al., 2014). Subsequently, WOX11/12 activates *WOX5/7* expression and confers the acquisition of regeneration competency by establishing root meristem fate (Atta et al., 2009; Sugimoto et al., 2010; Hu and Xu, 2016; Rosspopoff et al., 2017). Finally, cytokinin signaling components type-B ARABIDOPSIS RESPONSE REGULATORs initiate the expression of *WUSCHEL* (*WUS*), the master regulator of shoot meristem maintenance, and thus generate the shoot meristem (Meng et al., 2017; Zhang et al., 2017; Zubo et al., 2017). The interaction of auxin and cytokinin plays critical roles in *de novo* organogenesis through altering epigenetic modifications and controlling expression of key transcription factors (Li et al., 2011; Cheng et al., 2013; Ikeuchi et al., 2019).

Recent studies provided substantial insights for understanding *de novo* organogenesis (Ikeuchi et al., 2019; Williams and Garza, 2021). However, the difference of regulatory mechanisms between direct and indirect shoot regeneration remains unknown. Distinct culture conditions of these two regeneration types make the comparison difficult. In this study, we established a bi-directional regeneration system, in which shoots regenerated directly and indirectly simultaneously. The results based on this system revealed that callus generated more founder cells which express *WOX11* and gave rise to lager converting organs and shoot meristems. Both *WOX11* transcription and direct shoot regeneration were regulated by non-CG DNA methylation. The results suggest that non-CG DNA methylation play different roles in direct and indirect regeneration via modulating *WOX11* transcription.

## Materials and Methods

### Plant Materials and Growth Conditions

*Arabidopsis thaliana* ecotype Col-0 was used as the wild type in this study. The *gWUS-GFP3* reporter lines were kindly provided by Thomas Laux (University of Freiburg) (Tucker et al., 2008). The of *pARR1:ARR1-GFP* reporter lines have been described previously (Meng et al., 2017). The *WOX11pro:H2B-eGFP*reporter lines were kindly provided by Lin Xu (Chinese Academy of Sciences) (Zhai and Xu, 2021). The *drm1 drm2 cmt3-11* triple mutant was kindly provided by Xiaofeng Cao (Chinese Academy of Sciences) (Cao et al., 2003).

Seedlings were grown under sterile condition at 20–22°C, with 16 h of white light and 8 h of dark. Segments containing hypocotyl and root were used as explants, which were firstly germinated in GM medium containing 10 μM auxin transport inhibitors naphthylphthalamic acid (NPA), and then transferred onto the medium containing Gamborg’s B5 medium with 2% glucose, 0.5 g/L MES, 10 μM 1-naphthaleneacetic acid (NAA), and 0.8% agar. After 2 days culture, explants were transferred onto SIM containing Gamborg’s B5 medium with 2% glucose, 0.5 g/L MES, 9 μM 2-isopentenyladenine (2-iP) and 0.8% agar for shoot induction. Explants were cultured under full white light. For calculation of shoot regeneration frequency, regenerated tissues containing a meristem surrounded by three or more leaf primordia with a phyllotactic pattern were considered as a shoot.

### Explant Imaging and Analysis

Olympus SZX-16 stereoscopic microscope (Olympus) was used to observe explants during regeneration procedures. The expression signals of reporter lines were observed using low melting point agarose embedding section. Confocal microscopy images were taken using a Zeiss LSM 880 NLO confocal microscope with a 20 × lens. Multitracking in line scanmode and a 488/561main dichroic filter were used to image GFP and dsRED together (Heisler et al., 2005). A 561-nm laser line and a 600–640-nm band-pass filter were used for dsRED. A 488-nm laser line and a 505–550-nm band-pass filter were used for GFP. Cell outline was stained with Fluorescent Brightener. A 405-nm laser line and a 425–475-nm band-pass filter was used for observation.

### qRT-PCR

Total RNA was extracted using the TRIzol™ Reagent (catalog no. 15596-026, Invitrogen). The full-length cDNA was generated with the RevertAid First-strand cDNA synthesis kit (Thermo). qRT-PCR was performed on a Chromo4 real-time PCR system (Bio-Rad) using SYBR Master mix (Vazyme) with gene-specific primers (Supplementary Table 1). Transcript levels of the examined genes were normalized to that of the housekeeping gene tubulin2. Values shown are the mean ± standard deviation (SD) of three biological replicates.

### Bisulfite Sequencing Analysis

DNA was isolated using a cetyltrimethylammonium bromide method. DNA methylation assay was performed using DNA Bisulfite Conversion Kit (Tiangen). PCR products amplified with Methylation specific PCR kit (Tiangen) were cloned into Blunt3 vector (TransGen Biotech) and sequenced. Bisulfite sequencing data were analyzed by the CyMATE software. The results returned by CyMATE were put into GraphPad Prism 9.0 to illustrate DNA methylation frequency at CG, CHG and CHH (where H = A, C or T), respectively. Primers were list in Supplementary Table 1.

## Results

### Establishment of the Bi-Directional Regeneration System

**FIGURE 1:**
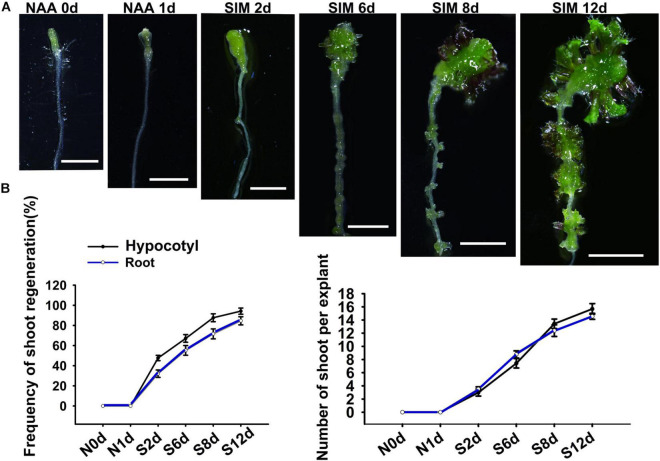
The bi-directional shoot regeneration system. **(A)** Explants exposed to NAA treatment or incubated in SIM at different days. Scale bars represent 20 mm. **(B)** Frequency of shoot regeneration and regenerated shoot number per explant. Error bars represent the standard deviations of three biological replicates. For each replicate, more than 50 individual plants were used.

In order to study the difference between regulatory mechanisms of direct and indirect shoot regeneration, we first tied to establish a bi-directional regeneration system in which shoots can be generated through the two pathways under the same culture condition. For this purpose, we used segments containing hypocotyl and root as explants, and modified a direct regeneration system reported previously by adjusting the hormone concentrations (Rosspopoff et al., 2017). The results show that when explants were treated with 10 μM NAA for 48 h and then cultured in shoot-inducing medium (SIM) containing 9μM 2-iP, shoots were regenerated from both the hypocotyl and the root (Figure 1). After 2 days incubation on SIM (SIM2), the hypocotyl produced callus while the root gave rise to protuberances. Subsequently, both callus and protuberance grew in size and produced shoot meristems at SIM6. At SIM8, leafy shoots were formed. Therefore, in this system, shoots were generated indirectly from hypocotyls and directly from roots simultaneously (Figure 1).

### Callus Produced Larger Shoot Meristems Than That of Lateral Root Primordia

**FIGURE 2:**
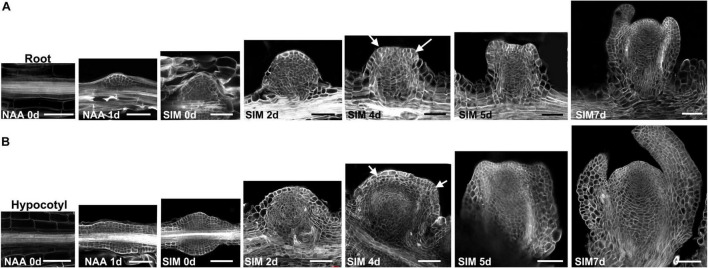
Cytological features of shoot regeneration procedures of the bi-directional system. **(A)** Shoots were regenerated through direct conversion from LRP into shoot meristem. **(B)** Shoots were produced indirectly from the hypocotyl-derived callus. Days after NAA-treatment or SIM-culture are indicated in the bottom left corner of each panel. Arrows point to the position of leaf primordia. Scale bars represent 50 μm.

We next compared the cytological features of these two regeneration procedures by observing their histological structures. Consistent with previous studies, NAA treatment promoted the formation of LRPs (Chatfield et al., 2013; Rosspopoff et al., 2017). After transfer to SIM, the LRP gradually grew into roundish converting organ based on cell divisions at multiple orientations (Figure 2A). Leaf primordia initiated at SIM4 and the structure of shoot meristem was established in the following 1–2 days. In comparison, exogenous NAA induced callus formation in hypocotyls (Figure 2B). After 1 day culture in SIM, the callus grew into a flattened structure. Compared with that of LRP at the same stage, the basal part of callus was much wider, which gave rise to converting organs and shoot meristems with significantly larger size in the subsequent stages.

### Callus Initiation Was Accompanied by More Founder Cells Expressing *WOX11* Than That of Lateral Root Primordia

**FIGURE 3:**
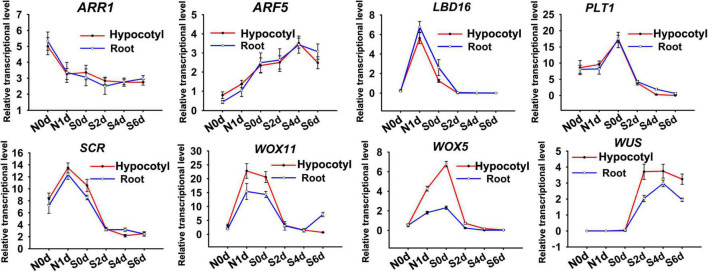
qRT-PCR analysis of genes involved in shoot regeneration. N represents days after NAA treatment. S indicates days for SIM incubation. Error bars show standard deviations of three biological repeats.

To investigate the cause of the different meristem size that regenerated from the two regeneration types, we examined the transcriptional levels of genes involved in shoot regeneration. The selected genes encode transcriptional factors regulating auxin/cytokinin signaling (*ARF5* and *ARR1*) or stem cell identity (*LBD16*, *PLT1*, *SCR*, *WOX5*, *WOX11*, and *WUS*). qRT-PCR revealed that transcriptional levels of *ARF5*, *ARR1*, *LBD16*, *PLT1*, and *SCR* exhibited similar dynamic patterns between direct and indirect regeneration procedures, suggesting their conserved roles in the two different regeneration pathways (Figure 3). Transcripts of *WUS* was not detectable during NAA-treatment stage. However, SIM-incubation caused obvious increase of *WUS* expression, which was more significant in the hypocotyl explants. Transcription of *WOX5* and *WOX11* was induced by exogenous NAA but decreased during SIM culture. The NAA-mediated alteration of *WOX11* and *WOX5* was more pronounced in hypocotyl explants than that in root. The results suggest that conversion of cell identity might be differently regulated between direct and indirect regeneration.

**FIGURE 4:**
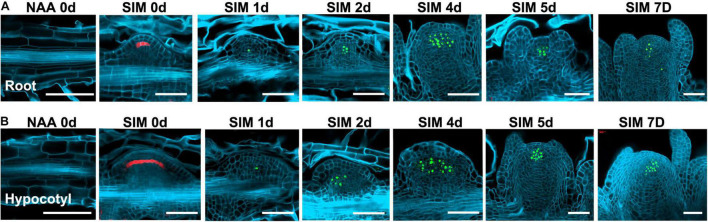
Expression signals of the *pWOX5:RFP; gWUS-3GFP* double reporter lines during shoot regeneration. **(A)** Procedure of direct regeneration through conversion from LRP into shoot meristem. **(B)** Indirect regeneration from the hypocotyl-derived callus. Days after NAA-treatment or SIM-culture are indicated on top of each panel. Scale bars represent 50 μm.

**FIGURE 5:**
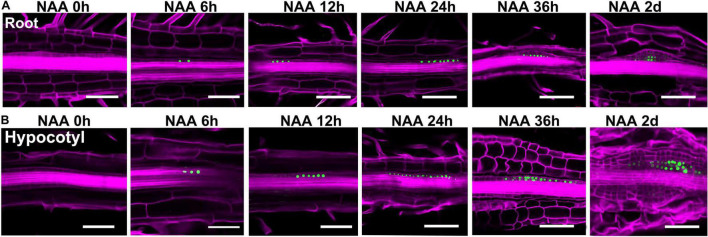
Expression patterns of *WOX11* revealed by the *pWOX11:H2B-eGFP* lines during shoot regeneration. **(A,B)** Illustrate direct and indirect regeneration processes, respectively. Hours or days for NAA-treatment are indicated on top of each panel. Scale bars represent 50 μm.

To get more insights into the cell fate transition process, we visualized the spatio-temporal expression signals of *WOX5*, *WUS*, and *WOX11*, respectively (Figures 4, 5). The *pWOX5:RFP; gWUS-3GFP* double reporter lines revealed that *WOX5* and *WUS* were expressed in similar patterns in hypocotyl and root explants (Figure 4). After 48 h NAA-treatment, *WOX5* was expressed in the middle cell layers in both callus and LRP. At SIM1, the expression signal of *WOX5* vanished while that of *WUS* was initiated in a few cells. As the callus and LRP grow in size, *WUS* expression expanded into larger domains. When shoot meristem was formed, *WUS* expression was confined to the organizing center. The most obvious difference between the two types of explants is that the expression domain of *WOX5* at the end of NAA-treatment in callus was larger than that in LRP.

In the *pWOX11:H2B-eGFP* lines, GFP signals were first detected in pericycle cells at 6 h of NAA-treatment (Figure 5). Different from that of LRP, where *WOX11* was induced in about 8 cells before periclinal division at 12 h, expression signals were visible in more than 15 continuous cells in the initiating callus. During the primary cell divisions, the signals were also observed in newly proliferated cells. When organized cell files were established, *WOX11* was expressed in the founder cells at basal part of the callus and LRP. Therefore, number of founder cells in the incipient stage was tightly associated with the regeneration types and the size of regenerated meristems. Relatively more *WOX11*-expressing founder cells gave rise to callus which produce larger meristem, whereas less founder cells led to the formation of LRP that regenerate smaller meristem.

### Non-CG DNA Methylation Regulates Direct but Not Indirect Shoot Regeneration

**FIGURE 6:**
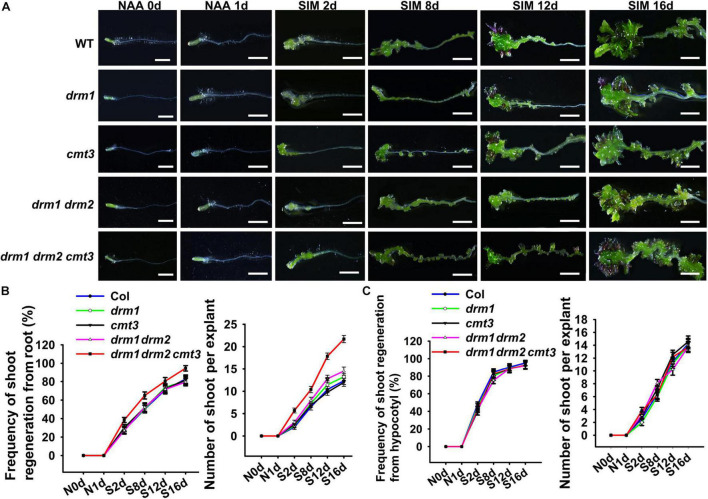
Comparisons of shoot regeneration capacity between the *ddc* triple mutant and the wild type. **(A)** Shoot regeneration of *drm1*, *cmt3*, *drm1 drm2*, and *ddc* mutants. Days after NAA-treatment or SIM-culture are indicated in the bottom left corner of each panel. Frequency of shoot regeneration and number of regenerated shoots per explant are shown for direct **(B)** and indirect **(C)** regeneration systems. The frequency of direct regeneration was calculated as the shooted hypocotyl number/total hypocotyl number, while that of indirect regeneration was determined as the shooted root number/total root number. Error bars indicate the standard deviations of three biological replicates. For each replicate, more than 50 individual plants were used. Scale bars represent 10 μm.

We next intend to explore the factors regulate *WOX11* expression. Previous studies showed that non-CG methylation is involved in acquisition of pluripotency (Shemer et al., 2015). It has been shown that non-CG DNA methylation is almost completely lost in the triple mutant of DOMAINS REARRANGED METHYLTRANSFERASE1/2 CHROMOMETHYLASE3 (*drm1 drm2 cmt3*) (Cokus et al., 2008; Stroud et al., 2014). We thus examined the shoot regeneration capacity of the *drm1 drm2 cmt3* (*ddc*) triple mutant using the bi-directional system described above. As a result, both the frequency and the number of regenerated shoots per explant were significantly increased in root of the *ddc* triple mutant compared with those of wild type (Figure 6). However, the regeneration ability of hypocotyl did not show obvious changes between the mutant and the wild type. The results indicate that non-CG DNA methylation negatively regulates direct shoot regeneration but did not affect indirect regeneration. No obvious phenotype was observed in *drm1* and *cmt3* single mutants, as well as *drm1 drm2* double mutant, suggesting the functional redundancy among *DRM1*, *DRM2* and *CMT3*.

### Non-CG DNA Methylation Mediated *WOX11* Expression and Lateral Root Primordia Formation

**FIGURE 7:**
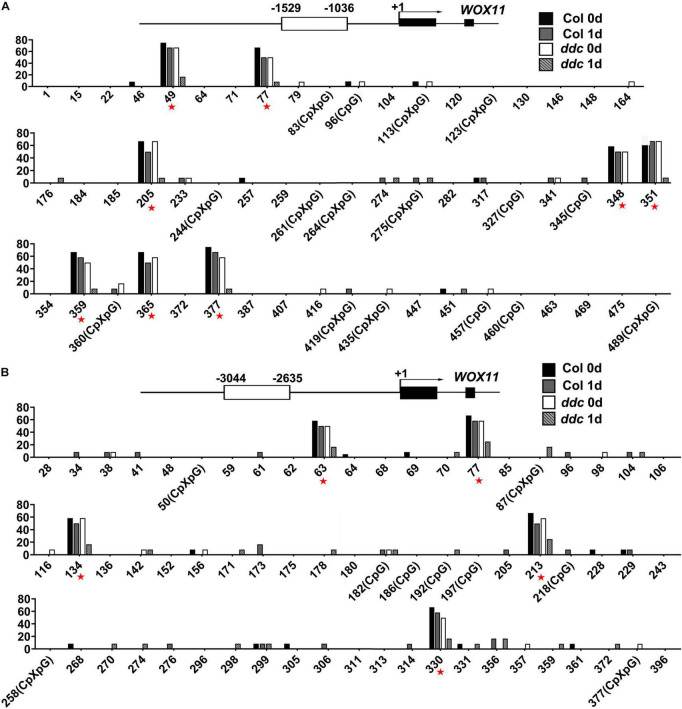
Analysis of methylation level in the promoter region of *WOX11* via bisulfite sequencing. Levels of cytosine methylation in genomic fragments 1,036–1,529 bp **(A)** and 2,635–3,044 bp **(B)** upstream of the coding sequence were detected. Root explants incubated under NAA-treatment were used for analysis. Red asterisk.

**FIGURE 8:**
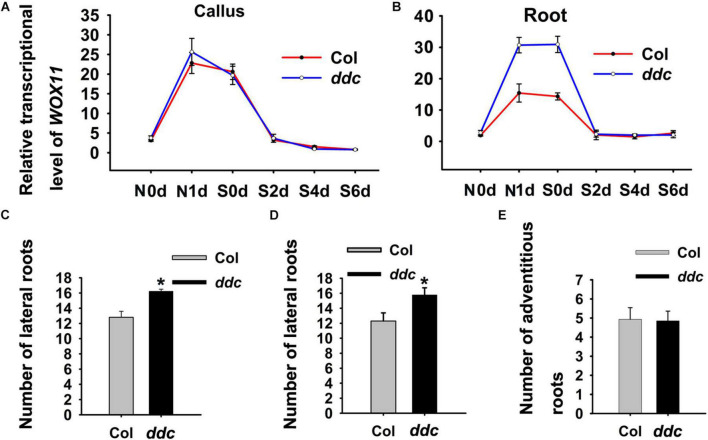
Dynamics of *WOX11* transcript levels in hypocotyl **(A)** and root **(B)** explants derived from *ddc* and wild-type seedings. **(C)** Lateral root numbers derived from *ddc* and wild-type seedlings grown on hormone-free medium. **(D)** Lateral root numbers of *ddc* and wild-type explants after 48 h NAA-treatment. **(E)** Adventitious root number of *ddc* and wild-type hypocotyls which were cultured in hormone-free medium after 48 h NAA-treatment. Error bars show standard deviations of three biological repeats. *0.001 < *P* < 0.01 are determined by two-tailed Student’s *t*-tests.

To determine whether the expression of *WOX11* is mediated by non-CG DNA methylation, bisulfite sequencing was performed to compare DNA methylation between the *ddc* triple mutant and the wild type. The results illustrate that after NAA-treatment for 1 day, 13 sites of the genomic fragments 1,036–1,529 bp and 2,635–3,044 bp upstream of the coding sequence were hypermethylated in wild type. However, the level of methylation in the same sites were substantially decreased in the *ddc* mutant (Figure 7). Correspondingly, compared with that of wild type, transcriptional level of *WOX11* was significantly higher in the *ddc* root during NAA-treatment (Figure 8A). On the contrary, in the hypocotyl explants at the same stages, both the methylation and the expression of *WOX11* did not show obvious difference between *ddc* and wild type (Figure 8B and Supplementary Figure 1). The results demonstrate that non-CG DNA methylation negatively regulate *WOX11* transcription during LRP formation but had no influence on callus.

*De novo* shoot regeneration comprises three steps, including the activation of initial cells, acquisition of regenerative competency and establishment of shoot meristem (Sang et al., 2018b). *WOX11* controls the former two steps by promoting the first cell fate transition and activating *WOX5* expression (Liu et al., 2014; Hu and Xu, 2016; Zhai and Xu, 2021). Subsequently, genes responsible for shoot meristem maintenance such as *WUS* regulate the third step. Therefore, if non-CG methylation regulate shoot regeneration via modulating *WOX11* expression, the *ddc* triple mutant would produce more LRP. To test this hypothesis, we examined lateral root number. The results show that the *ddc* triple mutant give rise to significantly more lateral roots than that of wild type, indicating an increase in LRP formation (Figure 8C).

Previous study showed that after transfer to hormone-free medium, auxin-induced callus which resembles LRP can be converted to roots (Atta et al., 2009). To analyze the difference of NAA-induced callus/LRP formation between the *ddc* triple mutant and wild type, we transferred explants after 48 h NAA-treatment to hormone-free medium. As a result, the *ddc* triple mutant generated significantly more lateral roots than wild type (Figure 8D). However, the number of adventitious roots derived from hypocotyls did not demonstrate obvious changes, suggesting that callus formation capacity was similar between the *ddc* triple mutant and wild type (Figure 8E). These results suggest that non-CG methylation is implicated in shoot regeneration through mediating *WOX11* expression.

## Discussion

Owing to its theoretical and practical importance, shoot regeneration have been substantially studied (Williams and Garza, 2021). It is well acknowledged that during culture in auxin-rich medium, explants from aerial or root organs give rise to callus, which subsequently generate shoots under cytokinin induction (Duclercq et al., 2011). Recent studies showed that exogenous cytokinin can directly convert LRP into shoot meristem (Atta et al., 2009; Chatfield et al., 2013; Kareem et al., 2015; Rosspopoff et al., 2017). Thus, the direct and indirect regeneration experienced distinct developmental programs, but the difference of their regulatory mechanisms remains elusive. Because the media formulations used in these two pathways are quite different, it is difficult to compare direct and indirect regeneration under the same condition. In the present study, we established a bidirectional system, in which shoots were produced directly from root and indirectly from hypocotyl synchronously, and thus provided a system for comparing the different regeneration pathways (Figure 1).

Using the bi-directional regeneration system, we analyzed the expression of homeodomain family genes that mark cell fate transition. Of them, the expression patterns of *WOX5* and *WUS*, which represent the identity of stem cell niches, were similar between the procedures of direct and indirect regeneration (Aichinger et al., 2012). Consistent with previous findings, *WUS* expression signal was initiated in only a few cells at early stage of cytokinin-incubation, and expended into larger domain afterward, indicating that fate transition from root meristem to shoot meristem is a gradual process (Figure 4; Meng et al., 2017; Zhang et al., 2017). *WOX11* activates the initial step for regeneration by priming founder cells, and is continuously expressed in the founder cells during callus formation (Liu et al., 2014; Zhai and Xu, 2021). In this context, perivascular cells expressing *WOX11* can be reckoned as stem cells, which produce new cells through proliferation and maintain their identity at the same time. Our results show that at the early stages of regeneration, perivascular cells with *WOX11* expressional signal were much more in callus than those in LRP, indicating that the “initiating site” for callus formation was relatively larger (Figure 5). Consistently, in the following stages, callus formed wider structure and generated larger converting organs and shoot meristems than that of LRP. The results suggest that the number of founder cells determines the manner of regeneration and the size of regenerated organ.

It has been revealed previously that *WOX11* is not expressed and not involved in LRP initiation from seedlings grown vertically on hormone-free medium (Sheng et al., 2017). However, when the primary root is damaged, *WOX11* expression is induced at the wounding site and mediates lateral root formation. The wound-induced lateral roots are completely inhibited by excision of aerial part and can be recovered by application of auxin at the decapitated region. The results suggest that the basipetal auxin transport is required for lateral root formation upon wounding by inducing *WOX11* expression. Therefore, it is plausible to infer that in the present study, exogenous NAA in the early culturing stage initiated *WOX11* expression and subsequent LRP formation.

Non-CG DNA methylation provided a conjunction that connected *WOX11* expression to shoot regeneration. In the *ddc* triple mutant, where non-CG DNA methylation is almost completely lost, direct regeneration was significantly promoted while indirect regeneration was unaffected (Figure 6). Correspondingly, the transcriptional level of *WOX11* was increased in *ddc* root compared with that of wild type, but was unchanged between *ddc* and wild-type hypocotyl (Figures 8A,B). Therefore, it is reasonable to speculate that DNA methylation-mediated *WOX11* expression was specifically implicated in the regulation of direct shoot regeneration. Callus formation resembles the root development pathway (Atta et al., 2009; Sugimoto et al., 2010). It is possible that callus derived from aerial organs was generated similar to adventitious root. Recent studies have revealed different regulatory mechanisms between the formation of adventitious and lateral roots (Bellini et al., 2014; Verstraeten et al., 2014). DNA methylation-mediated *WOX11* expression might be a specific factor for the latter.

Overall, our study compared direct and indirect shoot regeneration using the bi-directional system. The results revealed two lines of difference, both of which were mediated by *WOX11*. Firstly, number of founder cells that express *WOX11* determined the type of regeneration. Callus initiation was accompanied by more founder cells and regenerated larger organs, while less founder cells were established in LRP and gave rise to smaller meristems. Secondly, non-CG DNA Methylation specifically regulated *WOX11* expression and direct shoot regeneration, and had no influence on indirect regeneration.

## Data Availability Statement

The original contributions presented in the study are included in the article/Supplementary Material, further inquiries can be directed to the corresponding author/s.

## Author Contributions

ZC and YS conceived and designed the experiments. JL and WD performed the experiments and data analysis with the help of FF, XL, XHZ, and YZ. XSZ and YS wrote the manuscript. All authors contributed to the article and approved the submitted version.

## Conflict of Interest

The authors declare that the research was conducted in the absence of any commercial or financial relationships that could be construed as a potential conflict of interest.

## Publisher’s Note

All claims expressed in this article are solely those of the authors and do not necessarily represent those of their affiliated organizations, or those of the publisher, the editors and the reviewers. Any product that may be evaluated in this article, or claim that may be made by its manufacturer, is not guaranteed or endorsed by the publisher.

## Supplementary Material

Supplementary Figure 1 Methylation level in the promoter region of *WOX11* via bisulfite sequencing. Cytosine methylation levels in genomic fragments 1,036–1,529 bp **(A)** and 2,635–3044 bp **(B)** upstream of the coding sequence were detected. Hypocotyl explants incubated under NAA-treatment were used for analysis.
