# Supplementary figures and images for: Regulation of WOX11 Expression Represents the Difference Between Direct and Indirect Shoot Regeneration
Source: Front Plant Sci. 2022 Mar 4;13:850726. doi: 10.3389/fpls.2022.850726 (PMC8931721; doi:10.3389/fpls.2022.850726)

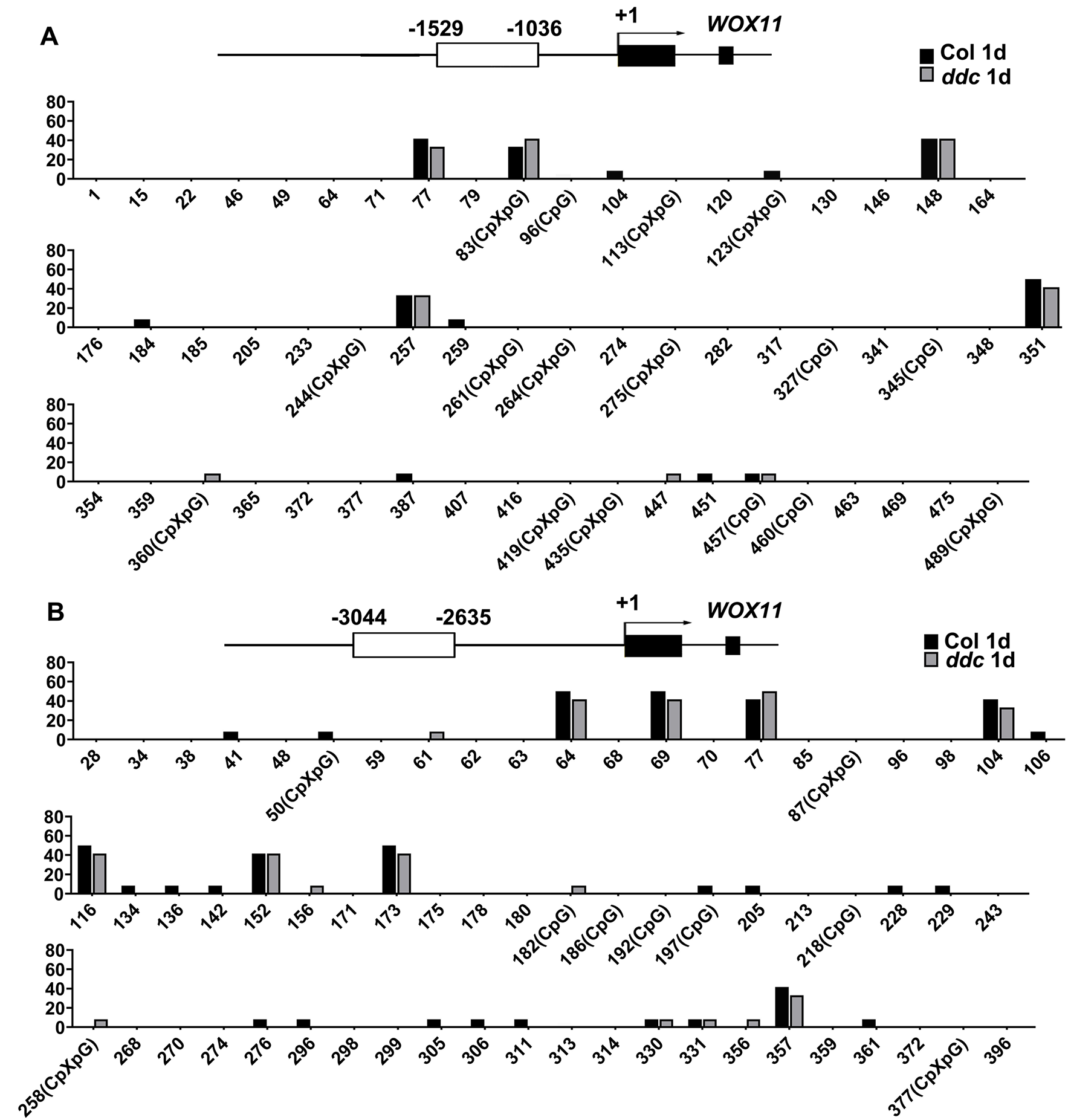

Supplement: Supplementary Figure 1 — Methylation level in the promoter region of WOX11 via bisulfite sequencing. Cytosine methylation levels in genomic fragments 1,036–1,529 bp (A) and 2,635–3044 bp (B) upstream of the coding sequence were detected. Hypocotyl explants incubated under NAA-treatment were used for analysis. [file Image_1.TIF]
